# The role of pulmonary mesenchymal cells in airway epithelium regeneration during injury repair

**DOI:** 10.1186/s13287-019-1452-1

**Published:** 2019-12-02

**Authors:** Suyun Fang, Suhong Zhang, Haiting Dai, Xiaoxiang Hu, Changgong Li, Yiming Xing

**Affiliations:** 1 State Key Laboratory for Agrobiotechnology, College of Biological Sciences, China Agricultural University, Beijing, People’s Republic of China; 2 National Engineering Laboratory for Animal Breeding, China Agricultural University, Beijing, China; 3 Department of Pediatrics, Division of Neonatology, University of Southern California, Keck School of Medicine, Los Angeles, CA USA

**Keywords:** Lung, *Dermo1*, Mesenchymal stem cell, Lipopolysaccharide, Naphthalene, Injury repair

## Abstract

**Background:**

The airways of mammalian lung are lined with highly specialized cell types that are the target of airborne toxicants and injury. Several epithelial cell types and bone marrow-derived mesenchymal stem cells have been identified to serve as stem cells during injury repair. However, the contributions of endogenous mesenchymal cells to recruitment, expansion or differentiation of stem cells, and repair and reestablishment of the normal composition of airway epithelium following injury have not been addressed.

**Methods:**

The role of mouse pulmonary mesenchymal cells was investigated by lineage tracing using *Dermo1-Cre*; *ROSA*^*mTmG*^ mice. In experimental models of lung injury by lipopolysaccharide and naphthalene, GFP-labeled *Dermo1*^*+*^ mesenchymal cells were traced during injury repair. In vitro lung explant culture treated with or without lipopolysaccharide was also used to verify in vivo data.

**Results:**

During injury repair, a subgroup of GFP-labeled *Dermo1*^*+*^ mesenchymal cells were found to contribute to normal repair of the airway epithelium and differentiated into Club cells, ciliated cells, and goblet cells. In Club cell-specific naphthalene injury model, the process of *Dermo1*^*+*^ stem cell regenerating epithelial cells was dissected. The *Dermo1*^*+*^ stem cells was migrated into the airway epithelium layer sooner after injury, and sequentially differentiated transitionally to epithelial stem cells, such as neuroendocrine cells, and finally to newly differentiated Club cells, ciliated cells, and goblet cells in injury repair.

**Conclusion:**

In this study, a population of *Dermo1*^*+*^ mesenchymal stem cell was identified to serve as stem cells in airway epithelial cell regeneration during injury repair. The *Dermo1*^*+*^ mesenchymal stem cell differentiated into epithelial stem cells before reestablishing various epithelial cells. These findings have implications for understanding the regulation of lung repair and the potential for usage of mesenchymal stem cells in therapeutic strategies for lung diseases.

## Introduction

Throughout life, multicellular organisms must regenerate cells to maintain the integrity and functions of their tissues after injury, but the capacity to repair the tissue damage may fail due to repeated injury and aging. The adult lung is one of the few organs that has a direct interface with the outside environment. The epithelial cells that line the airways are constantly exposed to potential toxic agents and pathogens. Therefore, it must be able to respond quickly and effectively to recover the cellular damage. The cellular hallmark of lung repair after injury is a rapid proliferative and differentiation response ultimately leading to restoration of the airway epithelium. Several origins of the stem cells that repair damaged airway epithelium have been identified [1–4]. However, precise information regarding their emergence and diversification during injury repair is scant.

The conducting airways of the mammalian lung are composed of three major epithelial cell types, namely ciliated cells, non-ciliated Club cells, and neuroendocrine (NE) cells. These cells are arranged in an orderly manner to form branches and alveolar structures [5]. The mesenchymal part is crucial in determining the shape and size of the lung, which can be subdivided into sub-mesothelial mesenchyme (marked by the expression of WNT2A and FGF10) and sub-epithelial mesenchyme (marked by the expression of NOGGIN) [6]. Pulmonary mesenchyme contains a host of complex cell lineages including lymphatics, endothelial cells, smooth muscle cells, myofibroblasts, cartilage-forming cells, and mesothelial cells [7]. The normal development of pulmonary mesenchyme is associated with the successful extension and branching of the airway [8, 9]. Coordination of pulmonary mesenchyme and epithelium is required to form a functional lung.

Various experimentally induced whole lung and airway injury models have been used, such as the toxicant naphthalene (NAPH) mediated abolishing of Club cells and the acute lung injury induced by lipopolysaccharide (LPS). Subsequently, the surviving cells are thought to serve as stem/progenitor cells to restore the epithelium. There are evidences that several cell types serve in this capacity. Fist, a subset of NAPH-resistant variant Club cells has been identified that seems to have the ability to self-renew as well as generate other cell types [3]. Second, the pulmonary neuroendocrine (NE) cells are distinguished mainly by expression of PGP9.5. NE cells reside within a unique microenvironment known as neuroepithelial bodies and undergo expansion subsequent to NAPH injury [10]. In bacterial LPS model, the lung parenchyma is damaged by the generation and release of proteases and reactive oxygen and nitrogen species produced by activated lung macrophages and transmigrated neutrophils in the interstitial and alveolar compartments. The end results are microvascular injury and diffuse alveolar damage with intrapulmonary hemorrhage, edema, and fibrin deposition [11]. Mesenchymal stem cells (MSCs), commonly referred to as adult marrow stromal cells, show capacity to differentiate into a number of mature cell types, including fibroblasts, myofibroblasts, and epithelial cells [12]. Treatment with MSCs protected LPS injured mice from death by decreasing the edema and reducing the inflammatory response [13]. Recent study observed significant number of labeled MSCs had migrated from the bone marrow to the lung lesions and differentiated to macrophages, alveolar epithelial cells, and interstitial fibroblasts and myofibroblasts [14]. As ongoing maintenance of the airways and repair after injury are key to normal respiratory function, precise knowledge of which cell type(s) are recruited to reestablish airway homeostasis and the precise mechanics of how repair is controlled is of significant interest.

Exogenously infused MSCs modulate tissue injury and repair. These properties have led to novel therapeutic strategies involving exogenous administration of MSCs in various injury and disease settings. Despite the broad therapeutic potential of this cell type, the in vivo role of endogenous MSCs remains undefined due to the absence of specific markers. In the current study, we demonstrate that a subgroup of *Dermo1*^*+*^ mesenchymal cells serve as MSCs to regenerate airway epithelial cells during LPS and NAPH-induced injury repair in mouse lung. These endogenous MSCs sequentially differentiated transitionally to epithelial stem cells, such as neuroendocrine cells, and finally to newly differentiated Club cells, ciliated cells and goblet cells. Moreover, the *Dermo1*^+^ MSCs are not HH/Gli1 signaling regulated mesenchymal cells.

## Materials and methods

### Mouse lines

*Dermo1-Cre*, *Gli1-Cre*^*ERT2*^, and *Gt (ROSA)26Sor*^*tm4(ACTB-tdTomato,-EGFP)Luo*^*/J* (referred as *ROSA*^*mTmG*^) mice were gifts from Parviz Minoo (University of Southern California, USA). *Dermo1-Cre*; *ROSA*^*mTmG*^ and *Gli1-Cre*^*ERT2*^; *ROSA*^*mTmG*^ mice were generated by crossing *Dermo1-Cre* and *Gli1-Cre*^*ERT2*^ with *ROSA*^*mTmG*^ mice, respectively. All animals were maintained on a 12-h light/dark cycle with ad libitum access to water and feed in individually ventilated units in the specific-pathogen-free facility. During the experiment, all procedures, care, and handling of animals were in accordance with the guidelines developed by Beijing Association on Laboratory Animal Care and were approved by China Agricultural University (SKLAB-2015-10).

### Tamoxifen administration

Tamoxifen (Sigma, USA) was dissolved in corn oil (Sigma, USA) at a concentration of 20 mg/mL. For lineage-tracing studies, *Gli1-Cre*^*ERT2*^; *ROSA*^*mTmG*^ mice received five continuous doses of 75 mg/kg bodyweight tamoxifen via intraperitoneal injection to induce CRE-mediated GFP expression. Injury was induced after 10 days of chasing.

### Injury treatments

Adult mice (8–12 weeks) were selected for injury with no gender distinction. For LPS injury, 20 mL/kg bodyweight avertin (Sigma, USA, 20 mg/mL) was intraperitoneally injected to anesthetize the mice. Five milligrams per kilogram bodyweight LPS (Sigma, USA, 1 mg/mL, PBS for control mice) dissolved in PBS (phosphate-buffered saline, pH 7.4) was intratracheally instilled via a 24-gauge venous indwelling needle and a 1-mL syringe. An extra of 0.8 mL of gas was supplied to flush the liquid uniformly into the more distal bronchioles. Mice woke up naturally and sacrificed at 1, 3, 5, 7, or 14 days post injury (DPI). For naphathalene injury, 300 mg/kg bodyweight NAPH (Sigma, USA, 30 mg/mL, corn oil for control mice) dissolved in corn oil was intraperitoneally injected. Mice were sacrificed at 1, 3, 5, or 7 DPI. Three to 5 mice were analyzed per injury stage. Each injury process was repeated over three times.

### RNA isolation and real-time quantitative polymerase chain reaction (qPCR)

Tissue RNAs were extracted by Qiagen RNeasy Mini Kit (QIAGEN, Germany) according to the handbook. One microgram of total RNAs was applied to synthesize the first-strand cDNAs by promega M-MLV Reverse Transcriptase (Promega, USA). Primers used for qPCR were designed via Primer3 software. Melting curve and amplification analyses were used to validate the primers. Quantification of targeted genes was performed on Roche LightCycler480 instrument with LightCycler 480 SYBR Green-based real-time qPCR kit reagents (Roche, Switzerland). PCR was conducted using the default thermal cycling parameter setting. Sample expression level was normalized to glyceraldehyde-3-phosphate dehydrogenase (*GAPDH*) expression.

### Tissue collection, fixation, and HE staining

The lung tissues were collected from mice around 2 months old and fixed in 4% PFA for 16 h in 4 °C. For paraffin sections, tissues were dehydrated by gradient ethanol solutions, embedded in paraffin. For frozen sections, tissues were dehydrated by gradient sucrose solutions, embedded in OCT. Tissue sections in 5 μm were prepared for hematoxylin-eosin (HE) staining and immunohistochemistry analysis.

### Histological analysis

Lung tissue sections were stained with hematoxylin and eosin for HE analysis. α-SMA (A5228, Sigma, US); CC10 (07-623, Meck Millipore, US); CD44 (ab25340), CD90 (ab3105), GFP (ab13970), MUC5AC (ab3649), and SMMHC (ab53219) were from Abcam; DESMIN (sc-271677), FOXJ1 (sc-53139), and PDGFA (sc-128) were from Santa Cruz; KI67 (Rm-9106-S0, Thermos Fisher); PGP9.5 (53772, Anaspec); SOX2 (3579) and SOX9 (82630) were from Cell Signaling Technology.

### Lung explant culture

Whole mouse lungs were dissected into small pieces and embedded into growth factor reduced BD matrigel matrix (BD Biosciences, USA), diluted 1:1 in DMEM/Ham’s F12 medium. After polymerization of the matrigel at 37 °C in a humidified incubator, lung explants were covered with culture medium (DMEM/Ham’s F12, 100 U/mL penicillin, 100 μg/mL streptomycin, 10% FBS) with or without 10 μg/mL LPS and cultured at 37 °C in a humidified incubator under 5% CO2 for 6 h.

### Cell counting and image analysis

Images were captured by an Olympus BX53 microscope under × 20 objective or Nikon A1 laser scanning confocal microscope under × 100 objective after immunostaining (Additional file 2). By ImageJ software, more than 10 random fields per section under × 20 objective were analyzed for cell quantification.

### Statistical analysis

The experimental data are presented as the mean ± SD and were analyzed by paired Student’s *t* test using SPSS21.0 software to compare the difference between samples. *P* value < 0.05 was considered significant.

## Result

### *Dermo1*^*+*^ mesenchymal cells modulated regeneration of airway epithelium after LPS injury

**Fig. 1:**
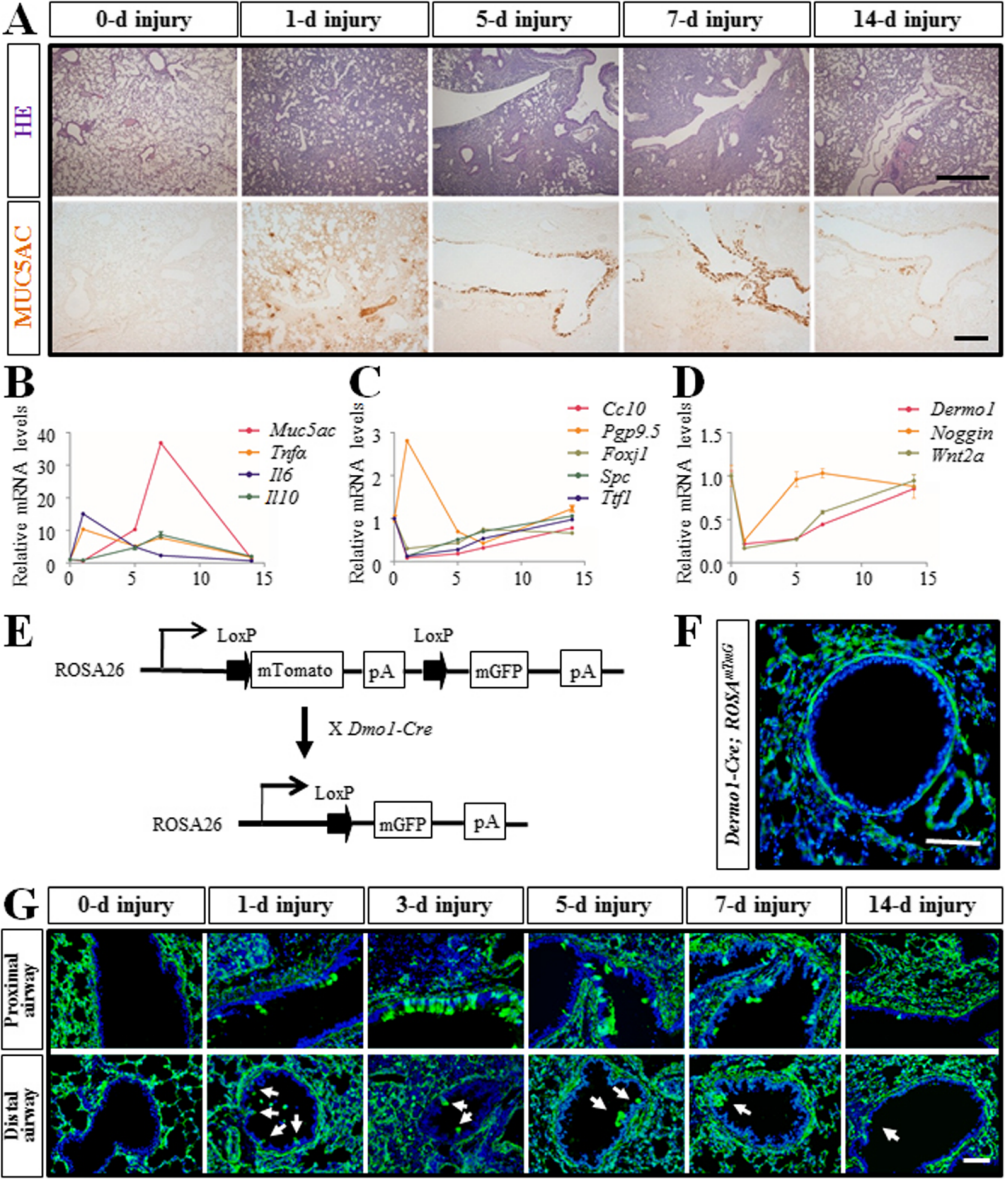
Generation of LPS injury model and *Dermo1-Cre>; ROSA*^*mTmG*^ mice. **a** HE staining and MUC5AC immunostaining respectively showed the inflammatory cells infiltration and the goblet cells hyperplasia during the injury-repair process after LPS injury. **b** Relative mRNA levels of inflammation related genes (*Tnfα*, *Il6*, and *Il10*) and mucin gene (*Muc5ac*) were verified by qPCR. **c** Relative mRNA levels of different pulmonary epithelial cell marker genes (*Cc10*, *Pgp9.5*, *Fo*xj1, S*pc*, and *Ttf1*) were verified by qPCR. **d** Relative mRNA levels of different pulmonary mesenchymal cell marker genes were verified by qPCR. **e** Schematic of *Dermo1-Cre; ROSA* ^*mTmG*^ mice. **f** Immunofluorescence staining showed GFP labeled pulmonary mesenchymal cells in *Dermo1-Cre*; ROSA^*mTmG*^ mice. **g** *Dermo*1^+^ lineage cells emerged into the airway epithelium layer after LPS injury. In the proximal airway, GFP^+^ cells were clustered while in the distal airway, GFP^+^ cells tended to be sporadic (arrow). d, days post LPS injury. Scale bar = 50 μm

The most commonly used experimental model for inducing acute lung injury is the bacterial LPS model. By intratracheal instillation of bacterial LPS, we induced severe pulmonary injury with inflammatory cell infiltration and goblet cell hyperplasia. MUC5AC belongs to the secreted mucin cluster and protects the mucosa from infection and chemical damage [15]. The result of MUC5AC antibody staining and HE staining showed that severe immunopathogenesis was induced at 1 day after LPS injection, and by 14 days, the lung was almost recovered (Fig. 1a). *Tnfα* encodes an inflammatory cytokine mainly secreted by macrophages. It is involved in the regulation of a wide spectrum of biological processes including cell proliferation, differentiation, and apoptosis [16]. *Il6* encodes a cytokine to induce fever after infections produced primarily at sites of acute and chronic inflammation [17]. *Tnfα* and *Il6* were highly expressed on 1 day after LPS injection and gradually decreased to healthy level during injury repair. IL10 is an anti-inflammatory cytokine secreted predominantly by monocytes that inhibit secretion of TNFα and IL6 induced by LPS [18]. The expression of *Il10* and *Muc5ac* were also largely increased after injection upon the inflammatory response (Fig. 1b). Various airway and alveoli derived epithelial cells were damaged after LPS challenge, such as Club cells, ciliated cells, and TypeII alveolar cells, the expression of those cell markers, *CC10*, *Foxj1*, *Spc*, and Ttf1, were also reduced respectively on day 1. In contrast, *Pgp9.5*, a marker of NE cells, was dramatically increased on day 1. By day 14, along with the cell recovering, the expression of these marker genes was restored as well (Fig. 1c). LPS injury also induced damage of mesenchyme-derived cells, including the condensed mesenchymal cells, the sub-mesothelial mesenchymal cells, and the sub-epithelial mesenchymal cells, as the expression of those cell markers, Dermo1, Wnt2a, and Noggin*,* were also reduced respectively on day 1 and restored on day 14 (Fig. 1d). Thus, by establishing the inflammatory injury model, we simulated the process of the pulmonary injury repair.

*Dermo1* is a basic helix-loop-helix transcription factor that is highly expressed in mesodermal tissues in mice [19]. To investigate whether lung endogenous mesenchymal progenitor/stem cells contribute to regeneration of the airway epithelium in injury repair, we have generated *Dermo1-Cre*; *ROSA*^*mTmG*^ mice by crossing *Dermo1-Cre* with *ROSA*^*mTmG*^ mice (Fig. 1e) [20]. In *Dermo1-Cre*; *ROSA*^*mTmG*^ mice, the *Dermo1* positive (*Dermo1*^*+*^) mesenchymal cells and all of their progeny express green fluorescent protein (GFP) (Fig. 1f). After LPS injection, *Dermo1*^*+*^ cells were observed on the epithelial layer in both proximal and distal airways on day 1. The number of *Dermo1*^*+*^ epithelial cells was increased on day 3 and decreased once the injury was repaired (Fig. 1g). The above results suggested that the *Dermo1*^*+*^ mesenchymal cells acted as stem cells to regenerate airway epithelium in LPS injury repair.

### *Dermo1*^*+*^ stem cells proliferated in airway epithelial cell regeneration after LPS injury

**Fig. 2:**
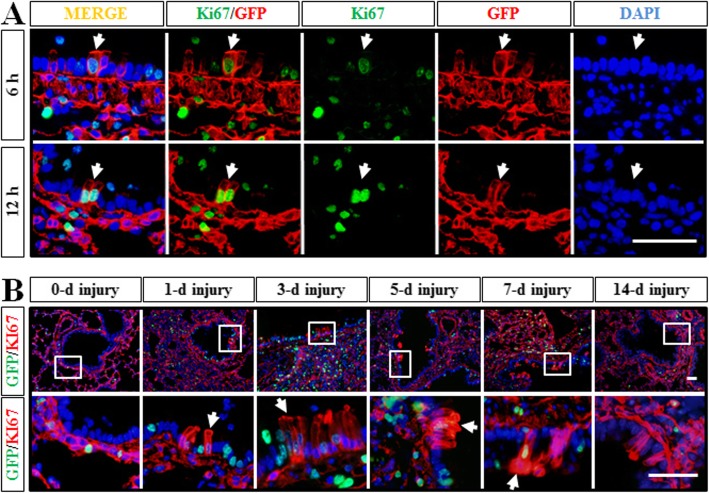
Proliferation analysis of *Dermo1*^*+*^ stem cells in LPS injury repair. **a** By Ki67 antibody staining, proliferating *Dermo1*^*+*^ stem cells were detected right after acute LPS injury at 6 h and 12 h. **b** *Dermo*1^+^ stem cells proliferated (arrow) to regenerate the airway epithelium in LPS injury repair. Isolated imagines of enlarged views of GFP^+^Ki67^+^ cells in the airway epithelium were indicated as white boxes. d, days post LPS injury. Scale bar = 50 μm

As described previously, we identified that a subgroup of *Dermo1*^*+*^ mesenchymal cells acted as stem cells to regenerate airway epithelial cells during LPS injury repair. Therefore, we further examined whether those *Dermo1*^*+*^ stem cells went to proliferate once migrated into the airway epithelial layer. By Ki67 antibody staining, proliferating *Dermo1*^*+*^ epithelial cells were detected as early as 6 h after LPS injection (Fig. 2a). On days 3 and 5, the proliferating *Dermo1*^*+*^ epithelial cells were found mostly aggregating as clusters, and they were hardly detectable on day 14 as the injury was almost repaired (Fig. 2b).

### *Dermo1*^*+*^ stem cells differentiated into various airway epithelial cell types in LPS injury repair

**Fig. 3:**
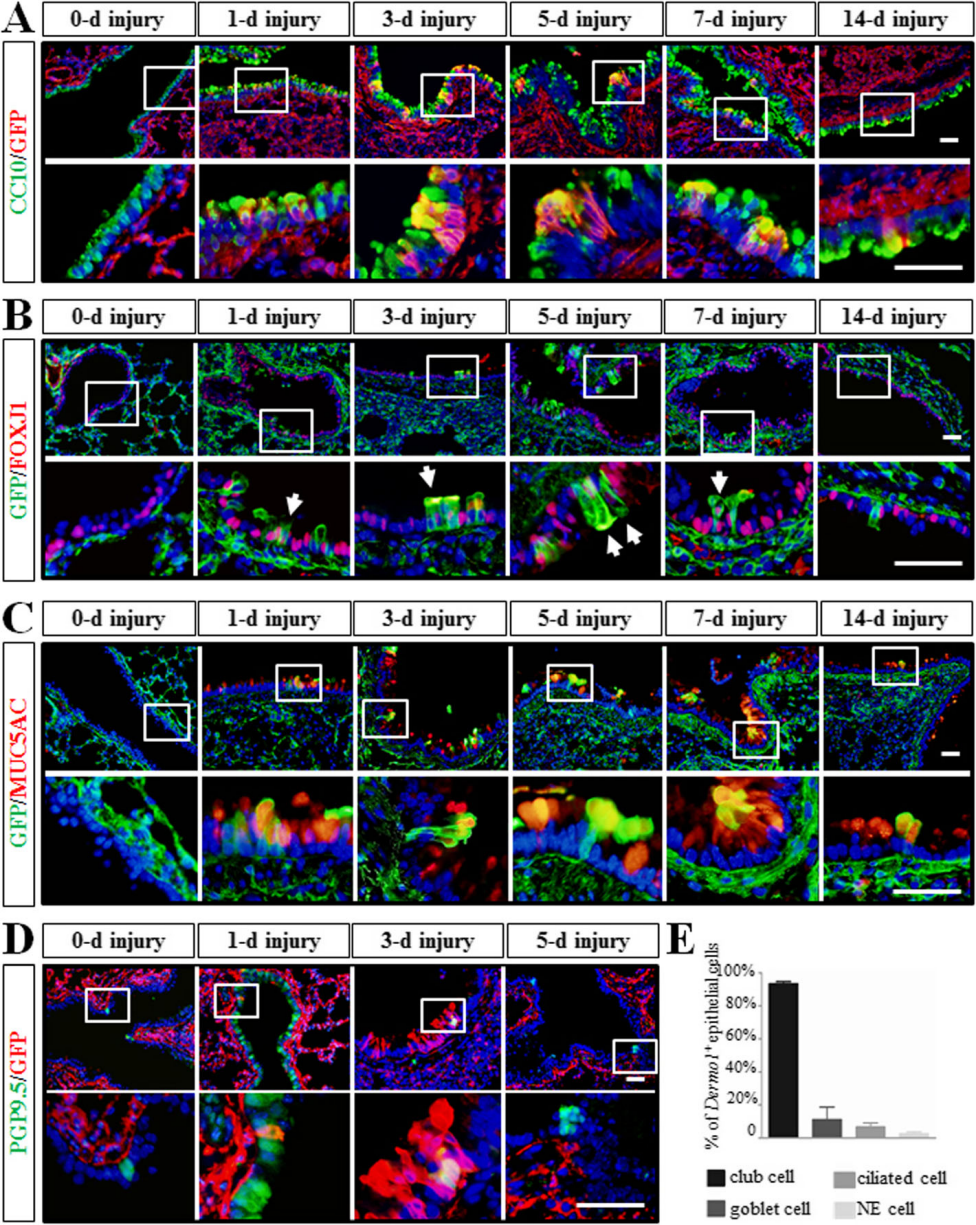
*Dermo1*^*+*^ mesenchymal stem cells regenerated diversified airway epithelial cells after LPS injury. *Dermo1*^*+*^ stem cells differentiated into Club cells (**a**), ciliated cells (**b**), goblet cells (**c**), and NE cells (**d**) after LPS injury. Isolated imagines of enlarged views were indicated as white boxes. **e** Statistics of *Dermo1*^*+*^ mesenchyme-derived Club cells, goblet cells, ciliated cells, and NE cells over total *Dermo1*^*+*^ stem cells in the proximal airway at 3 DPI. d, days post LPS injury. Scale bar = 50 μm

The major differentiated cellular constituents of the bronchial epithelium include Club cells, ciliated cells, basal cells, goblet cells, and NE cells. To determine which cell types were regenerated by *Dermo1*^*+*^ stem cells in LPS injury repair, the expression of cell-specific markers for Club, ciliated, goblet, and NE cells was examined by immunofluorescence. Antibodies to CC10, FOXJ1, MUC5AC, and PGP9.5 were used to double stained with GFP respectively. The overlaid expression pattern of the above markers and GFP indicated that *Dermo1*^*+*^ stem cells were able to give rise to Club cells, ciliated cells, goblet cells, and NE cells (Fig. 3a–d). Distribution of *Dermo1*^*+*^ stem cells in differentiating into each cell type was also examined by analyzing the rate of CC10^positive^, FOXJ1^positive^, MUC5AC^positive^, and PGP9.5^positive^ cells over total *Dermo1*^*+*^ stem cells. As the largest cell population in airway epithelium, the percentage of CC10^positive^/GFP^positive^ cells was 93.45% ± 2.52%, which was much higher than the percentages of FOXJ1^positive^/GFP^positive^, MUC5AC^positive^/GFP^positive^, and PGP9.5^positive^/GFP^positive^ cells (6.7% ± 4.63%, 11.19% ± 15.03%, and 2.56% ± 2.02%, respectively) at 3 days after LPS injury (Fig. 3e).

### *Dermo1*^*+*^ stem cells differentiated into airway epithelial cells after LPS treatment in vitro

**Fig. 4:**
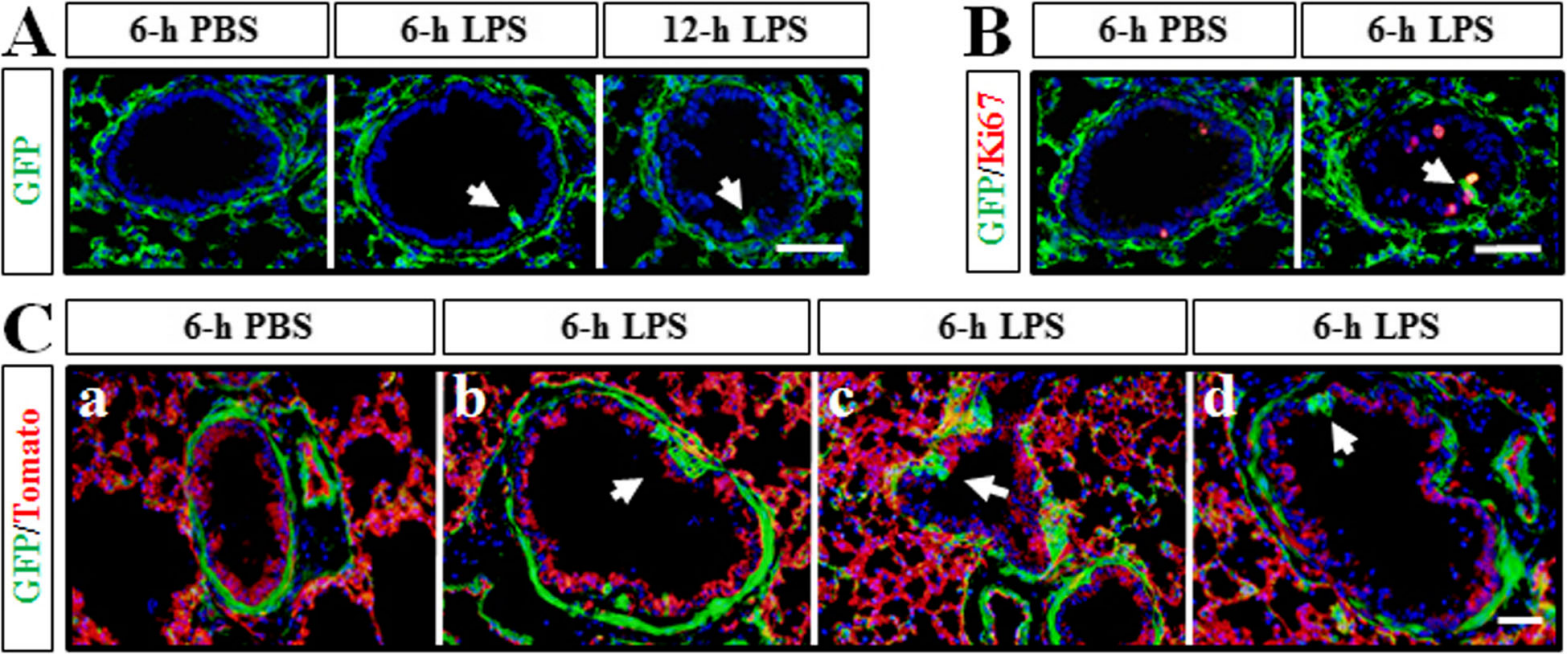
*Dermo1*^*+*^ stem cells regenerated airway epithelial cells in vitro after LPS treatment. **A** GFP antibody staining results showed that *Dermo1*^*+*^ cells emerged into the airway epithelium (arrow) at 6 h and 12 h after LPS supplementary in the culture medium. **B** Ki67 immunostaining results showed that proliferating *Dermo1*^*+*^ stem cells (arrow) were detected in the airway epithelium after 6 h of LPS treatment. **C** After 6-h treatment of LPS, *Dermo1*^*+*^ cells occurred in the airway epithelium with different migration-like forms (arrow) in frozen sections. Scale bar = 50 μm

For carefully investigating the movement of *Dermo1*^*+*^ stem cells in response to LPS, we performed a lung explant culture experiment. *Dermo1-Cre*; *ROSA*^*mTmG*^ mouse lung explants were dissected and cultured in Matrigel treating with or without LPS. After 6-h treatment, the *Dermo1*^*+*^ cells started to appear in the airway epithelial layer, which was consistent with previous in vivo results (Fig. 4A). Moreover, the *Dermo1*^*+*^ cells were proliferating after migrating into the epithelial layer (Fig. 4B). Interestingly, some clusters of *Dermo1*^*+*^ cells were also observed right under the epithelial cell layer (Fig. 4C (b)). The different locations of *Dermo1*^*+*^ cell clusters around the epithelial cell layer might simulate the movements of *Dermo1*^*+*^ stem cells in LPS injury repair.

### *Dermo1*^*+*^ stem cells regenerated Club cells through NE cells in NAPH-induced airway injury

**Fig. 5:**
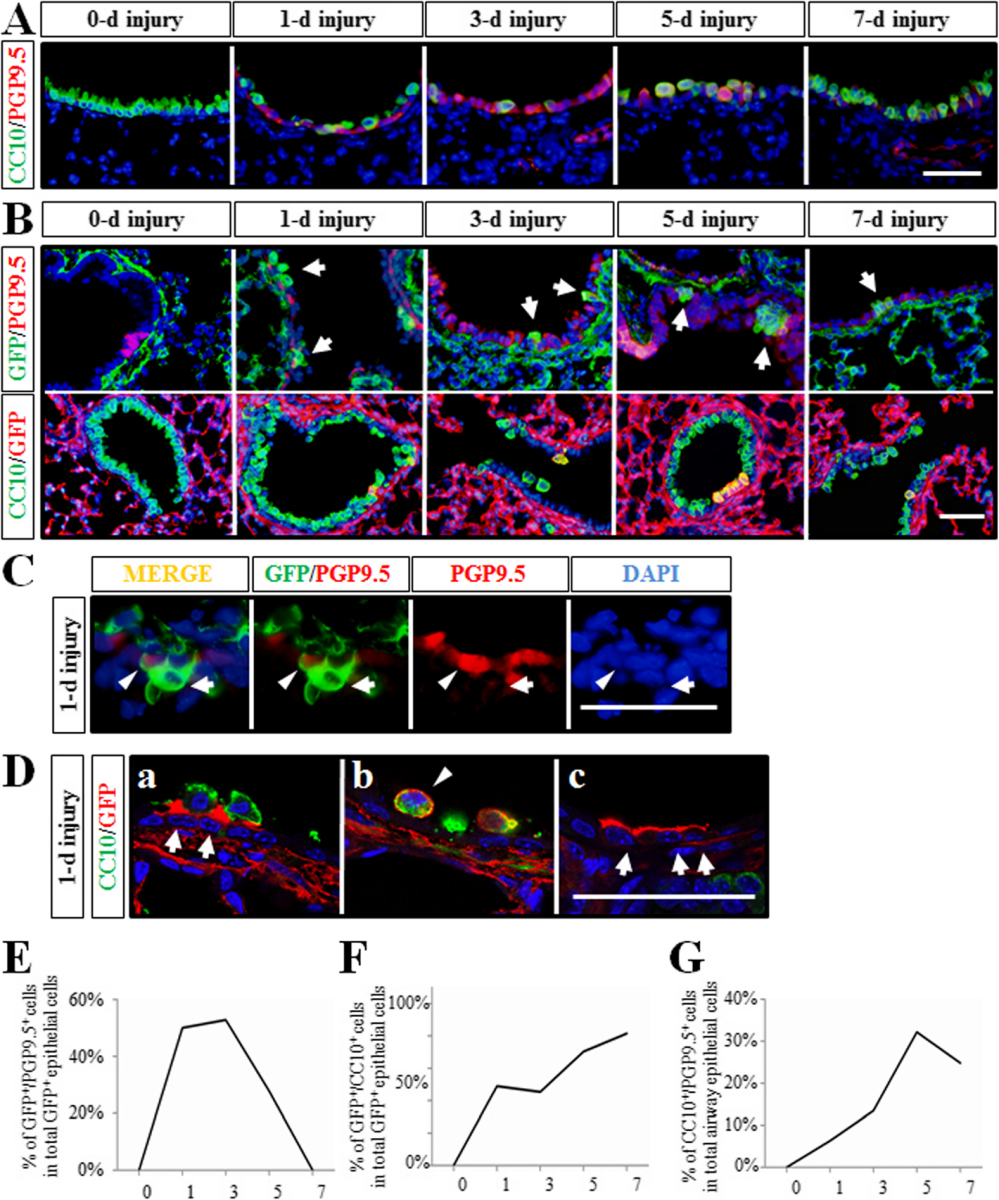
The process of *Dermo1*^*+*^ stem cells in Club cell regeneration in NAPH injury repair. **A** Establishment of NAPH injury model. Club cells were ablated gradually at day 1 and almost restored at day 7. NE cells increased and served as stem cell during Club cell regeneration. **B** *Dermo1*^+^ cells (arrow) appeared in the airway epithelium after NAPH injury displayed as GFP^positive^/PGP9.5^positive^ cells (upper panel) and GFP^positive^/CC10^positive^ cells (lower panel) respectively. **C** Amplified display of GFP^*+*^ NE cells (arrowhead) or non-NE cells (arrow) in the airway epithelium at day 1. **D** Both GFP^positive^/CC10^positive^ cells (arrowhead) and non-Club cells (arrow) in the airway epithelium were detected at day 1. **E** Dynamic changes of the proportion of CC10^positive^/PGP9.5^positive^ cells in the airway epithelium after NAPH injury. **F**, **G** Statistics of GFP^positive^/PGP9.5^positive^ (**E**) or GFP^positive^/CC10^positive^ (**F**) cells over total GFP^positive^ *Dermo1* stem cells after NAPH injury. d, days post NAPH injury. Scale bar = 50 μm

There is a significant body of data suggesting that NAPH-resistant Club cells and NE cells act as stem cells in airway regeneration after NAPH injury. In our previous work, we proved that NE cells are important source of stem cells to regenerate Club cells in NAPH injury repair [10]. Experimentally induced airway injury by NAPH is a commonly used injury model which abolishes Club cells. Subsequently, the surviving cells are considered to serve as stem cells to restore the bronchiolar epithelium. It deserved attention to identify whether *Dermo1*^*+*^ stem cells transdifferentiated into epithelial stem cells, such as NE cells, in Club cell regeneration. Thus, we explored 2-month-old *Dermo1-Cre*; *ROSA*^*mTmG*^ mice to NAPH (300 mg/kg body weight). Mazola corn oil was used as negative control. Lung tissues were analyzed on post-NAPH days 1, 3, 5, and 7. Antibodies to CC10, PGP9.5, and GFP were used to label Club, NE, and *Dermo1*^*+*^ cells respectively. Consistent with previous findings, by day 3 after NAPH treatment, the majority of CC10^positive^ cells were killed and released from the airway basement membrane, and the airway surface area was covered by PGP9.5^positive^ cells. On day 5 after NAPH exposure, newly regenerated CC10^positive^ cells comprised the airway epithelium. Subsequently, on day 7, the Club cell restoration was almost complete and the amount of the PGP9.5^positive^ and CC10^positive^/PGP9.5^positive^ cells were significantly reduced (Fig. 5A, G). By double-fluorescence immunostaining of GFP with CC10 or PGP9.5 respectively, we examined the role of *Dermo1*^*+*^ cell in Club cell regeneration. Interestingly, both GFP^positive^/PGP9.5^positive^ and GFP^positive^/CC10^positive^ cells were identified on day 1 after injury, which was earlier than the appearance of PGP9.5^positive^/CC10^positive^ (Fig. 5A–D). By analyzing the number of GFP^positive^/PGP9.5^positive^, GFP^positive^/CC10^positive^, and CC10^positive^/PGP9.5^positive^ cells, it confirmed that the presence of GFP^positive^/PGP9.5^positive^ cells started on day 1 and the number was increased on day 3. Alone with the airway recovery, the population of GFP^positive^/PGP9.5^positive^ cells was reduced on days 5 and 7 (Fig. 5E). Meanwhile, GFP^positive^/CC10^positive^ cells increased since day 1 and peaked at day 7 (Fig. 5). Thus, our analysis of dynamic changes in PGP9.5^positive^/*Dermo1*^*+*^ cell population subsequent to NAPH injury indicated a progression from NE/*Dermo1*^*+*^ cell to NE cell transitional cell intermediates and eventually newly regenerated Club cells. Since NAPH-resistant Club cells and NE cells are stem cells in CC10 regeneration, the above data suggested that the *Dermo1*^*+*^ mesenchymal stem cells differentiated into *Dermo1*^*+*^ epithelial stem cells after migrating into epithelial layer. Finally, the *Dermo1*^*+*^ epithelial stem cells terminally differentiated into new Club cells on days 5 and 7.

### *Dermo1*^*+*^ stem cells were not Gli1 signaling related

**Fig. 6:**
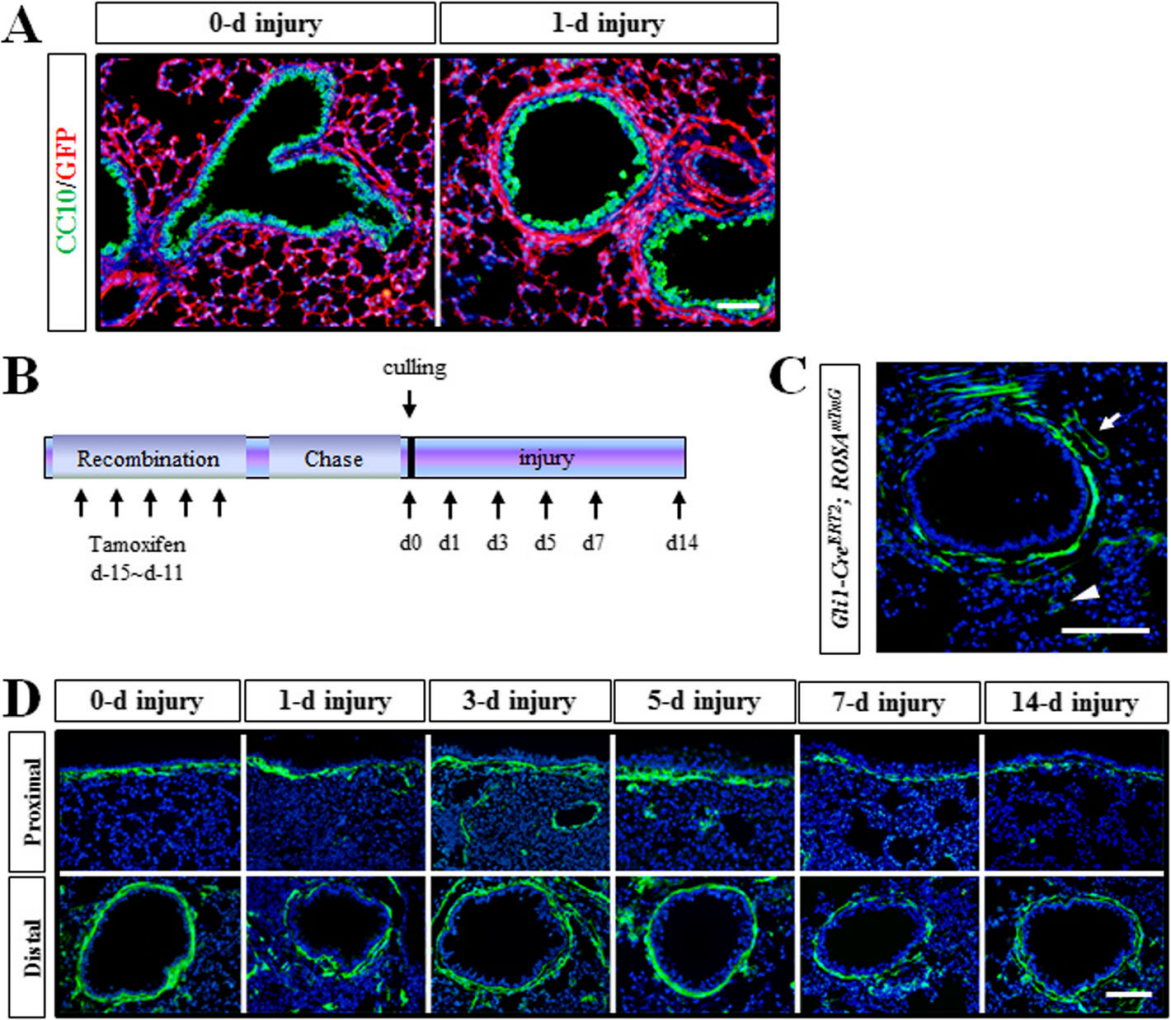
*Dermo1*^+^ stem cells were not Gli1 signaling related. **a** After NAPH injury, the pulmonary sub-epithelial mesenchymal layer was thickened in *Dermo1-Cre*; *ROSA*^*mTmG*^ mice. **b** Timeline of lineage tracing of *Gli1-Cre*^*ERT2*^; *ROSA*^*mTmG*^-labeled cells with tamoxifen treatment in NAPH injury repair. *Gli1-Cre*^*ERT2*^; *ROSA*^*mTmG*^ mice received five continuous dose of tamoxifen via intraperitoneal injection. Single dose of NAPH injury was conducted following 10 days of chasing. Lung tissues were collected at days 1, 3, 5, or 7. **c** Immunofluorescence staining results showed that GFP-labeled cells were mainly in the sub-epithelial and the peri-vascular (arrow) mesenchymal area in *Gli1-Cre*^*ERT2*^; *ROSA*^*mTmG*^ mice. A few of GFP^+^ cells were also detected in the sub-meshothelial mesenchyme (arrowhead). **d** Gli1^+^ cells were detected in airway epithelium after LPS injury. d, days post NAPH injury. Scale bar 50 μm

Recent work on the role of *Gli1*^*+*^ pulmonary cells has revealed the importance of SHH-Gli1 in regulating a feedback loop to maintain a balance between proliferation and quiescence during lung homeostasis and regeneration. GLI1 is expressed predominantly in mesenchymal cells adjacent to the proximal airway and pulmonary artery. In our studies, we have found the sub-epithelial mesenchymal layer which was next to the airway epithelium appeared significantly thickened after NAPH injury (Fig. 6a). Therefore, *Gli1-Cre*^*ERT2*^; *ROSA*^*mTmG*^ mice were generated to lineage trace *Gli1*^*+*^ cells in adult lung. Induction of CRE-mediated GFP expression labeled *Gli1*^*+*^ cells mainly located surrounding the airway epithelium and vessels. Only a spot of GFP^+^ cells scattered in the sub-mesothelial mesenchyme (Fig. 6b, c). The *Gli1-Cre*^*ERT2*^; *ROSA*^*mTmG*^ mice were exposed to LPS, and the behavior of *Gli1*^*+*^ cells was traced by GFP expression. Surprisingly, the *Gli1*^*+*^ cells appeared in the airway epithelium during LPS-induced injury repair (Fig. 6d). Therefore, the SHH-Gli1 signaling was not required for the function of *Dermo1*^*+*^ mesenchymal stem cells in epithelium regeneration.

### *Dermo1*^*+*^ stem cells were endogenous mesenchymal stem cells

**Fig. 7:**
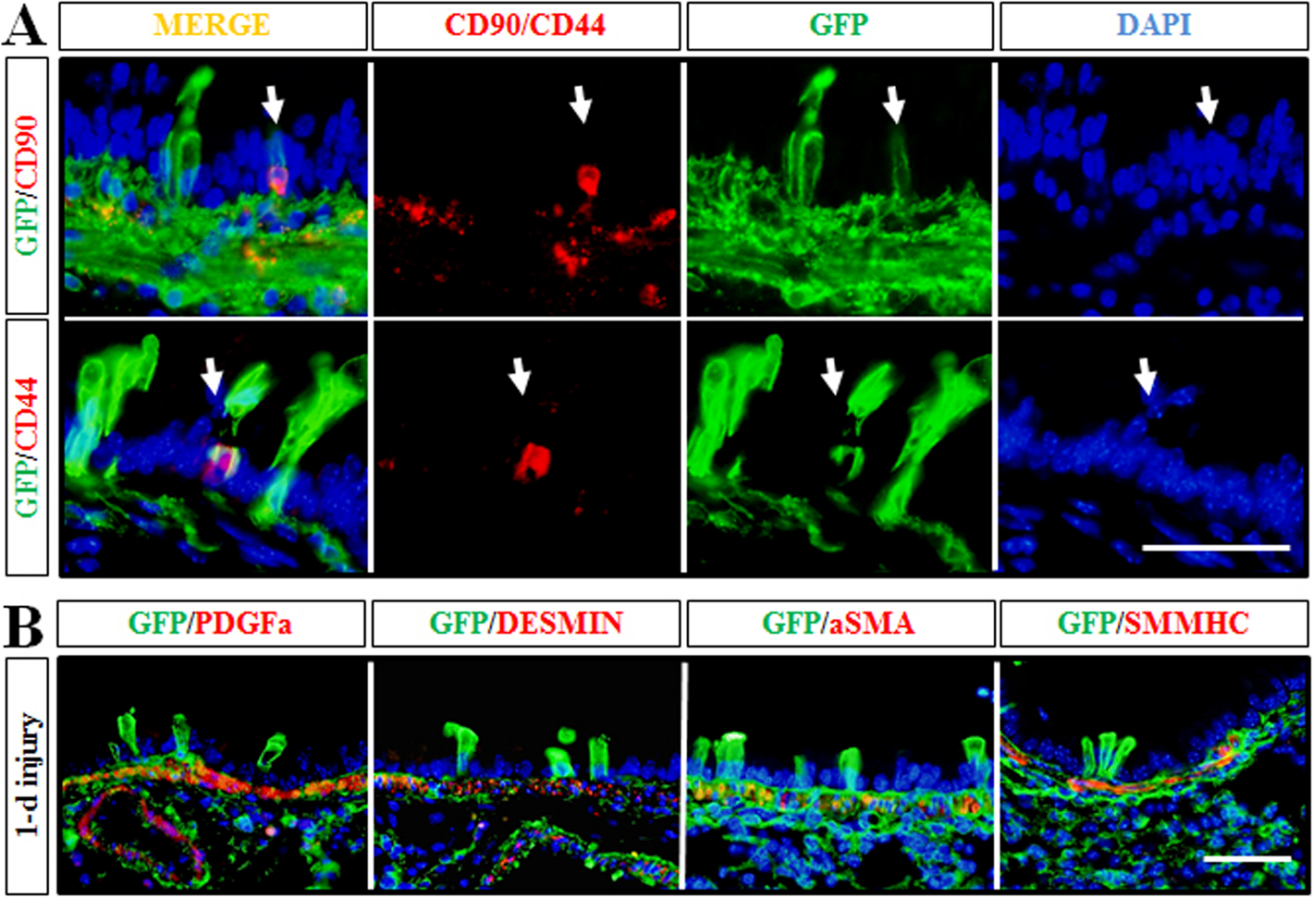
Analysis of *Dermo1*^+^ stem cells. **a** Very few *Dermo*1^+^ lineage stem cells in the airway epithelium were positive stained for mesenchymal stem cell markers, CD90 and CD44, after LPS injury. **b** *Dermo*1^+^ lineage stem cells in the airway epithelium were negative stained for general mesenchymal markers after LPS injury, including PDGFA, DESMIN, aSMA, and SMMHC. Scale bar = 50 μm

A simple plausible cause of the presence of *Dermo1*^*+*^ cells in the airway epithelial layer after injury may be bone marrow-derived stem cells through circulating blood. To examine this possibility, we used antibodies to CD44 and CD90, markers of bone marrow-derived mesenchymal stem cells, in *Dermo1-Cre*; *ROSA*^*mTmG*^ lungs exposed to LPS. Expression of CD44 and CD90 was found in very few *Dermo1*^*+*^ cells throughout the airways at day 3 after LPS exposure (Fig. 7a).

To address whether the *Dermo1*^*+*^ epithelial cells occurred in injury repair maintained characteristic of mesenchymal cells, the expression of pulmonary mesenchymal cell markers, PDGFA, DESMIN, αSMA, and SMMHC, were analyzed in *Dermo1*^*+*^ epithelial cells. In multiple experiments, we detected no expression of any of these genes in *Dermo1*^*+*^ epithelial cells (Fig. 7b).

## Discussion

The purpose of the current study was to determine the precise role of endogenous mesenchymal stem cells in lung injury repair. The choice of *Dermo1* was based on its specific expression in the pulmonary mesenchyme. In *Dermo1-Cre; ROSA*^*mTmG*^ mice, *Dermo1*^*+*^ cells were lineage traced by GFP labeling. Examination of airway epithelial regeneration in the LPS- and NAPH-induced injury/repair model revealed a process of *Dermo1*^*+*^ cell population dynamics characterized by expression of epithelial cell markers that lead to regeneration of Club cells, ciliated cells, and goblet cells. During this well-orchestrated process, *Dermo1*^*+*^ mesenchymal stem cells (MSCs) sequentially transdifferentiate into epithelial stem cells and terminal differentiated epithelial cells. These studies provide novel evidence that a subgroup of *Dermo1*^*+*^ cells serves as endogenous pulmonary MSCs in the re-establishment of a functional airway epithelium by regulating the step-wise transition of putative stem cells to transitional epithelial stem cell intermediates and, finally, to newly differentiated epithelial cells.

Lung mesenchyme is a critical determinant of the shape and size of the lung, the extent and patterning of epithelial branching, and the formation of the pulmonary vasculature and interstitial mesenchymal components of the adult lung [21]. Compared with epithelium, the composition of the pulmonary mesenchyme is more complicated. Crosstalk between pulmonary mesenchyme and epithelium has been demonstrated during lung development and injury repair. Different mesenchymal progenitor/stem cells have been identified to provide the niches for epithelia to maintain their stem cell capacity. For example, Axin2^+^Pdgfrα^+^ mesenchymal cells serve as alveolar stem cell niche supporting alveolar cell growth and regeneration, and Axin2^+^Pdgfrα^−^ myofibrogenic progenitors contribute to pathologically deleterious myofibroblasts [22]. Lgr6^+^ mesenchymal cells are also found to promote epithelial progenitors differentiate into airway cells via Wnt-Fgf10 cooperation, whereas Lgr5^+^ cells promote epithelial progenitors differentiate into alveolar cells through Wnt pathway [23]. Meanwhile, whether MSCs can differentiate into epithelial cells in injury repair is wildly studied. BMMSCs have shown the capability to ameliorate lung injury and generated a significant amount of interests as a potential therapy for acute or chronic lung diseases [24, 25]. MSCs work through multiple mechanisms, including cell engraftment, immunomodulation, alveolar fluid clearance, lung protein permeability, and antibacterial properties [26]. However, a number of issues central to whether or not using stem cells in therapeutic approaches will be successful. Problems with interpreting the results of these studies include having limited controls, failure of the stem cells to engraft, and the generation of inflammatory events by the treatment itself. In addition, the processing procedures, like isolation, in vitro amplification, immunological analysis [27], and uncontrollable change of cell morphology [24], will greatly limit their wide clinical applications. Thus, more and more tissue-resident endogenous mesenchymal stem cells are studied in a variety of tissues or organs, such as fat [28], placenta and blood [29], and skeletal muscle [30].

**Fig. 8:**
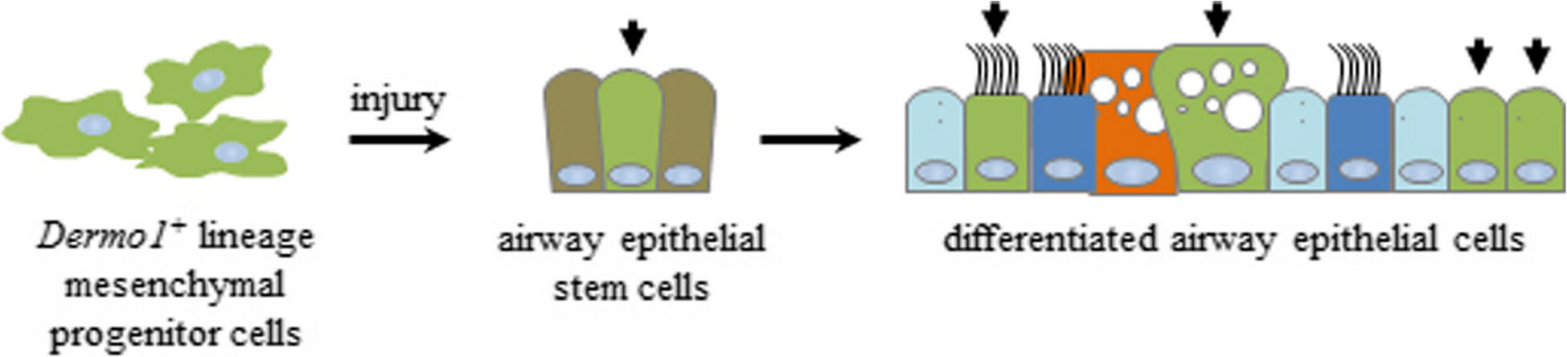
Schematic diagram of the mechanisms that *Dermo*1^+^ mesenchymal progenitor cells repaired the damaged airway epithelium. *Dermo1*^*+*^ mesenchymal stem cells firstly differentiated into epithelial stem cells and then regenerated the damaged epithelial cells via epithelial stem cells

The lung endogenous MSCs are first reported in 2007 that plastic-adherent fibroblastoid cells isolated from bronchoalveolar lavage fluid in patients undergoing lung transplant surgery are identified as MSCs [31]. The studies related to lung endogenous MSCs are carried out, most of which are implemented in vitro. In vivo studies showed minimal MSC activities which cannot exclude the result of auto-fluorescence of immunofluorescence reaction, and the therapeutic benefit is likely triggered by a paracrine-mediated mechanism [26]. Due to the lack of cell-specific markers, the understanding of the cellular interrelationships and dynamics between MSCs and epithelial cells in airway injury repair is emerged from robust cell lineage analyses. In our current study, by lineage tracing *Dermo1*^*+*^-GFP cells, we discovered that a subgroup of *Dermo1*^*+*^ cells served as stem cells in LPS- and NAPH-induced lung injury repair. Those endogenous mesenchymal stem cells migrated into the airway epithelium layer and transdifferentiated into epithelial stem cells, which in turn repaired the damaged epithelial cells, such as Club cells, ciliated cells and goblet cells (Fig. 8). Statistical analysis of the histological preparations in NAPH injury repair indicated that both GFP^positive^/PGP9.5^positive^ and GFP^positive^/CC10^positive^ cells appeared on day 1 after injury, which was earlier than PGP9.5^positive^/CC10^positive^ (Fig. 5). The data suggested that *Dermo1*^+^ MSCs migrated into the airway and transdifferentiated into epithelial stem cells, such as NE cells, and in turn regenerated the damaged epithelial Club cells. Previous studies also discovered that airway basal and Club cells serve as the primary progenitors of ciliated, goblet, and alveolar epithelial cells [32, 33]. Thus, we examined whether *Dermo1*^+^ MSCs transdifferentiated into basal cells during LPS injury repair. By P63 and GFP antibody double staining, we did not find GFP^positive^/p63^positive^ cells on day 1 after LPS injury (Additional file 1: Figure S1). Since the majority of GFP^positive^ cells were CC10^positive^, it is possible that *Dermo1*^+^ MSCs might differentiate into “club stem cells (or variant club cells)” during LPS injury. Meanwhile, the GFP^positive^/Foxj1^positive^ and GFP^positive^/MUC5AC^positive^ population did not increase from day 3 to day 14 (data not show); the possibility of Dermo1^+^ MSCs-Club stem cell-ciliated or goblet cell differentiation remains low. However, due to the lack of specific markers, “Club stem cells” or other type(s) of epithelial stem cells *Dermo1*^+^ MSCs transdifferentiated into is a query that had not been hitherto addressed.

The crosstalk between pulmonary epithelium and mesenchyme was widely reported. Among which, Shh signaling pathway played an important role. *Gli1*^*+*^ cells traced by Shh signaling pathway reporter mice acted as progenitor cells and promoted repair of the damaged epithelial cells through paracrine actions [34]. *Gli1*^*+*^ cells were peri-bronchial mesenchymal cells anatomically closer to the epithelial cell layer, which were also a subgroup of *Dermo1*^*+*^ cells. By tracing *Gli1*^*+*^ mesenchymal cells, surprisingly, *Gli1*^*+*^ cells did not contribute to the regeneration of airway epithelial after LPS injury. We realized that the current data could not distinguish which signaling pathways activated the *Dermo1*^*+*^ MSCs. The detailed mechanisms and the factors involved in required further investigation.

Acute lung injury (ALI) is an important problem in humans; however, its pathogenesis is poorly understood. To investigate the molecular mechanisms of ALI, various experimental models have been used, the most common being the endotoxin (bacterial LPS) model. In our study, intratracheal instillation of high dose of LPS caused severe lung injury as the inflammatory reaction was still intense at 7 days after injury. Various epithelial and mesenchymal derived cells were extensively damaged. Interestingly, the emergence of *Dermo1*^*+*^ MSCs in the airway epithelium was found in 1 day after injury. The quick response of *Dermo1*^*+*^ MSCs also occurred in the NAPH-induced injury model, which was even earlier than the presence of epithelial stem cells. Based on our observations, activation of endogenous mesenchymal stem cells, such as *Dermo1*^*+*^ MSCs, maybe a more preferable therapeutic approach than engrafting of bone marrow-derived MSCs.

## Conclusion

In sum, the results of this study suggest that, during mouse lung LPS and naphthalene injury repair, a population of *Dermo1*^*+*^ mesenchymal cells serve as a reservoir for epithelial cell regeneration and re-establishment of the normal airway epithelium. The *Dermo1*^*+*^ mesenchymal stem cell differentiated into epithelial stem cells before reestablishing various epithelial cells. These findings have implications for understanding the regulation of lung repair and the potential for usage of mesenchymal stem cells in therapeutic strategies for lung diseases.

## Supplementary information

**Additional file 1:** **Figure S1.** *Dermo1*^+^ stem cells did not transdifferentiated into basal cell in LPS injury repair. GFP and P63 antibody staining results showed that *Dermo1*^*+*^ stem cells did not labeled by P63 in airway epithelium at 6 h and 1 day after LPS injection. Scale bar = 50 μm.

**Additional file 2: Figure S2.** *Dermo1*^+^ stem cells were not SOX2 or SOX9 positive cells in LPS injury repair. By immunostaining, the result showed that *Dermo1*^*+*^ stem cells did not express Sox2 and Sox9 in airway epithelium at 1 and 3 day after LPS injection. E14.5 wild type lung was used as positive control. Scale bar = 50 μm.

## Data Availability

All data generated or analyzed during this study are included in this published article.

## Abbreviations

LPS: Lipopolysaccharide
MSC: Mesenchymal stem cell
NAPH: Naphthalene
NE: Neuroendocrine
