# Supplementary figures and images for: The role of pulmonary mesenchymal cells in airway epithelium regeneration during injury repair
Source: Stem Cell Res Ther. 2019 Dec 2;10:366. doi: 10.1186/s13287-019-1452-1 (PMC6889622; doi:10.1186/s13287-019-1452-1)

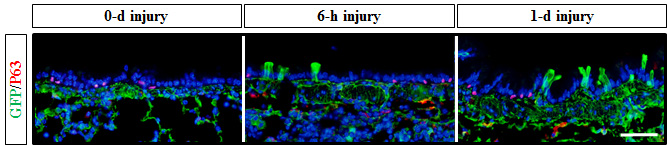

Supplement: Supplementary file 1 — Additional file 1: Figure S1. Dermo1+ stem cells did not transdifferentiated into basal cell in LPS injury repair. GFP and P63 antibody staining results showed that Dermo1+ stem cells did not labeled by P63 in airway epithelium at 6 h and 1 day after LPS injection. Scale bar = 50 μm. [file 13287_2019_1452_MOESM1_ESM.jpg]

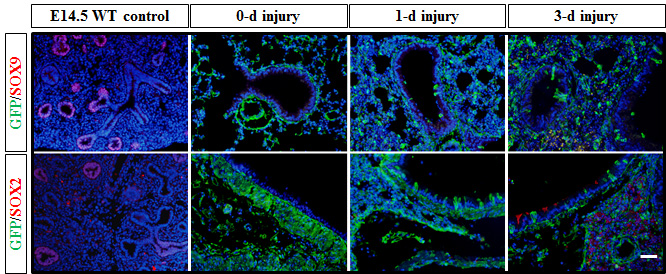

Supplement: Supplementary file 2 — Additional file 2: Figure S2. Dermo1+ stem cells were not SOX2 or SOX9 positive cells in LPS injury repair. By immunostaining, the result showed that Dermo1+ stem cells did not express Sox2 and Sox9 in airway epithelium at 1 and 3 day after LPS injection. E14.5 wild type lung was used as positive control. Scale bar = 50 μm. [file 13287_2019_1452_MOESM2_ESM.jpg]
